# Supplementary material for: Cullin 1 (CUL1) Promotes Primary Ciliogenesis through the Induction of Ubiquitin-Proteasome-Dependent Dvl2 Degradation
Source: Int J Mol Sci. 2021 Jul 15;22(14):7572. doi: 10.3390/ijms22147572 (PMC8307194; doi:10.3390/ijms22147572)
Supplement: Supplementary file 1 [file ijms-22-07572-s001.zip › ijms-1291592-supplementary/Supplemental information_Kim et al-KHL-submitted.pdf]

SUPPLEMENTAL INFORMATION

Cullin 1 (CUL1) promotes primary ciliogenesis through the induction of ubiquitin-proteasome-dependent Dvl2 degradation

Sun-Ok Kim, Kyoung Sang Cho, Bo Yeon Kim*, and Kyung Ho Lee*

* Corresponding author. E.mail: leekh@kribb.re.kr; bykim@kribb.re.kr

SUPPLEMENTAL FIGURE LEGENDS

Supplemental Table S1. Potential candidates of Dvl2 interacting proteins by Mass-spectrometry analyses. (Excel file)

Supplemental Table S2. List of siRNA sequences used in this study.

Oligonucleotides	Source	Cat. NO
Control siRNA-A	SantaCruz, Santa Cruz, CA	sc-37007
siCUL1 #1 (5′-GACGAAGGACGAAAAGGAATT-3′)	Bioneer, Daejon, South Korea	This study
siCUL1 #2 (5′-CAUUUUGGCGCAAGUUUUATT-3′)	Bioneer, Daejon, South Korea	This study
siCUL1 #3 (5′-CUAAACUUCAGCGCAUGUUTT-3′)	Bioneer, Daejon, South Korea	This study
CUL1 siRNA (h)	SantaCruz, Santa Cruz, CA	sc-35126

Supplemental Table S3. List of anti-bodies used in this study.

Antibodis	Host Species	Dilution	Source	Cat. NO
anti-Dvl2	Rabbit	1:1000 (IB)<br>1:50 (IF)	Cell Signaling, Danvers, MA	#3224S
anti-DVL2	Mouse	1:100 (IF)	Sigma, St. Louis, MO	SAB1405735
anti-CUL1	Mouse	1:1000 (IB)<br>1:100 (IF)	SantaCruz, Santa Cruz, CA	sc-17775
anti-Cullin 1	Rabbit	1:1000 (IB)	Abcam, Cambridge, MA	ab75817
anti-acetylated α-Tubulin	Mouse	1:200 (IF)	Sigma, St. Louis, MO	T7451
anti-γ-Tubulin	Rabbit	1:200 (IF)	Sigma, St. Louis, MO	T5192
anti-γ-Tubulin	Mouse	1:200 (IF)	Sigma, St. Louis, MO	T6557
anti-α-Tubulin	Rabbit	1:2000 (IB)	Abcam, Cambridge, MA	ab15246
anti-Flag	Mouse	1:1000 (IB)	Sigma, St. Louis, MO	F1084
anti-c-Myc	Mouse	1:1000 (IB)	SantaCruz, Santa Cruz, CA	sc-40
anti-HA probe	Mouse	1:1000 (IB)	SantaCruz, Santa Cruz, CA	sc-7392
Normal mouse IgG			SantaCruz, Santa Cruz, CA	sc-2025
Normal rabbit IgG			SantaCruz, Santa Cruz, CA	sc-2027

Supplemental Table S4. List of chemicals used in this study.

Chemicals and Reagents	Source	Cat. NO
X-tremeGENE HP DNA transfection reagent	Roche	06 366 546 001
Lipofectamine RNAiMAX reagent	Invitrogen	13778-150
pNPP (p-nitrophenyl phosphate)	Sigma, St. Louis, MO	N3254-100G
complete, EDTA-free protease inhibitor cocktail tablets	Roche	05 056 489 001
Phsophatase inhibitor cocktail 3	Sigma, St. Louis, MO	P0044
Fluoro-Gel (with Tris buffer) mounting solution	EMS	17985-10
Protein A-Agarose	SantaCruz, Santa Cruz, CA	sc-2001
Protein G PLUS-Agarose	SantaCruz, Santa Cruz, CA	sc-2002
DAPI	Sigma, St. Louis, MO	D9564
MLN4924, NAE inhibitor	Calbiochem	5.05477.0001
MG132	Calbiochem	474790
ALLN, Calpain inhibitor I	Sigma, St. Louis, MO	A6185
Lactacystin	Sigma, St. Louis, MO	L6785
Bafilomycin A1	Sigma, St. Louis, MO	B1793
Chloroquine	Sigma, St. Louis, MO	C6628
